# Aberdeen and Its Institutions

**Published:** 1892-10-08

**Authors:** 


### The Institutional Workshop.

### WITHIN THE HOSPITALS.

### ABERDEEN AND ITS INSTITUTIONS.

(By Our Special Commissioner.)

Aberdeen, apart from its geographical situation, from the fact that it possesses a university, a general, a children's, an incurable, a convalescent, and a fever hospital, in addition to a general dispensary, is an ideal community for a capable administrator to organise, so far as medical relief is concerned, upon a system, so sound and comprehensive, as to constitute an example for all similar cities in the Empire. On a bright day the visitor cannot fail to be struck with the beauty of Union Street, and he must have gone far to find a rival to it amongst other Scotch cities had the Town House been built across it at the market place end. The architectural and general features of Union Street favourably impress everyone; and the absolute purity and balmy character of the air during many months of the year prove most invigorating and pleasant. We gathered from the relatively limited hotel accommodation—which, however, we understood, was going to be considerably added to shortly—that British and American tourists have not yet found their way in any numbers to this beautiful city. Yet there is no place in Scotland better calculated to restore and recuperate exhausted nature, whilst the excursions to be made from it are so attractive, and the fishing to be obtained is so excellent, that it has only to become better known to ensure an influx of visitors throughout the greater part of each year. Aberdeen has one great advantage over most other Scotch cities, in the fact that there is very little squalor to be met with, the overwhelming majority of the inhabitants being thrifty and independent. We were further informed, upon reliable authority, and we can quite believe it, that publishers find that their books command a greater number of readers in Aberdeen than they do in any other British city of the same population. All these facts go to prove that Aberdonians have a right to claim that their city is ideal in many respects, and we hope, as there is good reason to anticipate, that the citizens will make it wholly ideal, so far as its medical institutions are concerned.

#### The Royal Infirmary.

The largest and best known of the medical institutions is the Royal Infirmary, established so long ago as 1739. Until seven years ago the internal condition of this institution, structurally, hygieneally, morally, left much to be desired. At this crisis Miss R[illegible]hel Frances Lumsden, who had done splendid service at the Children's Hospital, was invited, and consented to accept the office of Lady Superintendent. By her own wish the post was made honorary. The structural difficulties, which included very inadequate accommodation for the resident staff, were taken in hand, and after much anxious consideration, having regard to the contiguity of the Medical School, it was decided to appoint architects, and to rebuild the Infirmary, with the exception of the administration building, upon the old site. On the whole, we are inclined to think, although any change would have involved a considerable expenditure of money, that this decision was wrong. While it has in its favour the fact that the present site is the most convenient one available for the patients and their friends, other counterbalancing disadvantages outweigh in our view this consideration. However, reconstruction having been determined upon, and one entire wing having been rebuilt, it is too late in the day to treat this aspect of the question as a practical one. The reconstruction scheme included the keeping of the old building for administration and adaptation as a residence for nurses and other members of the staff. On the ground at the back of the administration building two large pavilions were to be built. Of these one has been completed, and at the time of our visit was being prepared for occupation, H.R.H. the Princess Louise having agreed to open it on October 4th. As the erection of this wing necessitated the pulling down of several of the old wards, it was impossible, having due regard to the claims of the suffering poor, to attempt to reconstruct the old central building (which remains in its original state at the present time) until after the new buildings were erected and occupied. In consequence, during the whole of the last seven years, Miss Lumsden has had to face this difficulty connected with the hygienic and moral condition in which she found the Infirmary. Thus handicapped, the work she undertook was enough to appal the most enthusiastic and able woman. It is, therefore, to her lasting honour that she has succeeded in removing many of the hygienic, and all the moral evils, and that she has organised a nursing staff, so intelligent and efficient as to make it compare favourably with that of any other large hospital. Her difficulties, we imagine, have been increased from the circumstance that the wages of the nurses and probationers, namely, £10 the first year, £14 the second and third, and £20 per annum afterwards, are lower than those, for example, at Dundee, or at any large infirmary in Scotland with which we are acquainted. She has fulfilled her task by confining the training to nurses, while she has imported the Sisters, all of whom are ladies, from the south. No language would be too strong in which to paint the service which Miss Lumsden has rendered to the City of Aberdeen, its citizens, and its poor. We have no doubt that the directors will take steps to raise the rate of remuneration paid to nurses, so as to bring it into line with other institutions, and to secure that each certificated nurse shall in future begin at £25 a year, rising to £30. They will also, we have every confidence from the record of intelligent service which stands already to their credit, promptly arrange a scheme which will, whilst it contracts them out of all further liability in regard to pensions, secure to each member of their staff a reasonable provision against the day of incapacity or old age.

#### The Children's Hospital.

The Children's Hospital owes its establishment to the devoted labours of Miss Lumsden, and to the influence and devotion of Dr. Robert Garden and his brother, the late lamented Mr. James M. Garden, who acted as Honorary Treasurer from the commencement. It consists of an old building converted, of a new wing of three storeys, and of a fever house, formerly a chapel, which contains two excellent wards, one for measles and the other for fever cases. The work of conversion and reconstruction has been skilfully carried out. The wards are, on the whole, surprisingly well lighted and airy, and if two beds were taken out of each of the wards situated in the converted house, their hygienic condition would leave little, if anything, to desire. The lavatories and bath-rooms, which contain fittings of an improved pattern, are disconnected from the wards, airy, and on the whole satisfactory. The amount of good work which this institution does for the children of Aberdeen is incalculable, and it is supplemented by a convalescent branch in the country, to which many little ones are received for change of air. At the present time the hospital is under the management of Miss K M. Lumsden, a sister of the lady who holds the post of Honorary Lady Superintendent to the Royal Infirmary.

#### The Hospital for Incurables and General Dispensary.

The Aberdeen Hospital for Incurables occupies one of the best and most beautiful sites in Aberdeen. It is a new and well proportioned building to which a male wing has recently been added. The wards are all airy, light, and most of them command extensive views from the windows. The sanitary and other fittings and furniture, and the general arrangements as well as the administration of this hospital make it one of the best, if not the best hospital for incurables in Great Britain. It is under the superintendence of a Matron, Miss Duguid, who was trained at the Edinburgh Royal Infirmary. She was formerly Matron at a similar institution in Ireland, and appears to be well qualified for the duties of her present office. It is a remarkable fact, seeing the urgent need that exists in this country to-day, that more beds for incurable cases are not available. Although the arrangements at this institution are excellent, and the good done is immense, yet the income for the last year, for which the accounts are forthcoming, only amounted to £695, leaving a deficiency of £800 to be made good. Such a state of things is remarkable, and, considering the (*a*) efficiency of this Home for Incurables, the fact (*b*) that it contains at the present time several vacant beds, and (*c*) the need which undoubtedly exists for such accommodation as it offers, we can only conclude that if its work be made widely known, funds will pour in, and many a poor soul now greatly afflicted will be materially comforted. Paupers, lunatics, idiots, epileptics, and persons suffering from blindness only, or who are at the point of death, are ineligible for admission. It further appears that applicants must be natives of the county of Aberdeen. We would suggest a repeal of this last regulation, and the institution should be thrown open to the whole country. This would lead to a great increase of the income, and might indefinitely extend the good which the institution undoubtedly does at the present time. The General Dispensary relieves persons resident within the dispensary district, which includes Aberdeen and its immediate neighbourhood. The medical work is done by a staff of non-resident medical officers, who receive a small sum for their services.

### Two Hard Cases.

It may be interesting to note that one of the cases at the Hospital for Incurables was that of a girl, aged seventeen, who was sent across the mountains to service in June about four years ago, was overtaken by a snow-storm, and lay for four days in a snowdrift. When she was found she was alive, but both feet were gangrenous. Her legs were amputated at the knee joint, and although her life was endangered for a long time, she is now able to walk on her knees, and is in good general health. She is a most capable seamstress, and it would be helpful to her, and cheering also, if some ladies in and around Aberdeen would so arrange that the poor girl be kept constantly supplied with work at a remunerative rate. Another sad case we saw at Aberdeen was that of a Sister at the Children's Hospital, who has contracted prurient ophthalmia from a patient, has lost the sight of one eye, and is threatened with the loss of the other. These cases forcibly bring home to one the uncertainties of life, and should make us all realise that at any time it may be our turn, if not to be similarly afflicted, at any rate to incur dangers which may result in permanent injury or loss of life.

### What Remains to be Done.

The medical wards and the re-construction of the administration building at the Royal Infirmary have yet to be undertaken. This fact will enable the directors to take steps to secure that they contain everything of the most modern and complete kind, and that they shall be so constructed as to attract experts from all parts of the kingdom, because they will then find in Aberdeen all that is best adapted and most suitable for hospital purposes. At the present time out-patients are not treated at the Infirmary if they are resident within the dispensary district. In other words, the managers of the Infirmary have had the wisdom to provide that the out-patient work shall be undertaken by the Dispensary so as to prevent any overlapping or abuse, and in return they pay to the Dispensary authorities £160 to £180 per year from the Burnett Fund. If the dispensary officers consider any case of sufficient importance they send it on to the Infirmary, where it receives the special treatment it may prove to be in need of. The City Corporation have a fever hospital under their management, for which they have recently voted about £10,000 in order to make it complete, and to extend it sufficiently to meet the needs of the whole population. Quite recently a movement has been set on foot in Aberdeen to establish a Nurses' Institute, apart from the existing institutions. We believe this to be a mistake, and would earnestly recommend that steps be taken to provide that the district nurses be worked from the General Dispensary, the buildings of which, we were informed, might readily be enlarged so as to contain sufficient accommodation. If this step be taken, having regard to the health properties of the splendid air of Aberdeen, we hope the managers of the Royal Infirmary will see their way to provide sufficient accommodation in the reconstructed administration building to establish an airy and comfortably-furnished Nurses' Home, for the reception of a large number of probationers, who could then be trained within its walls. If it is impossible to provide sufficient accommodation for a large nurses' training school in this way, then we would suggest that the Infirmary authorities should concert measures to raise a fund by issuing bonds at 4 per cent., the interest upon which they would guarantee. The interest and principal of the bonds should then be made a first charge on the earnings of the private nursing staff. The principal would be repaid by annual drawings, and thus in Aberdeen, as at other places, in a few years the whole of the buildings would be secured to the Infirmary. The capital sum raised by the issue of 4 per cent. bonds should be devoted to the erection, on a site adjacent to the Infirmary, of a Nurses' Home of the very best description. We are glad to see that the managers are already alive to the importance of providing a private nursing staff in connection with the Infirmary, as a part of its mission of service to the public. Miss Lumsden, whose proved capacity for the work is a material factor of the situation, must have an assistant in her present work when the new buildings are completed, and the sooner such an assistant is appointed the better. This would leave her hands free to work out the details of the suggestions here made, and we do not doubt that the result will be not only memorable in the history of Aberdeen, but satisfactory to everybody concerned. The authorities of the Children's Hospital, too, might usefully approach the City Corporation with a view to securing an adequate provision at the City Fever Hospital for children suffering from scarlet fever, measles, and other infectious diseases This would enable them to save the expense attending the maintenance of their present fever house; it would free the inhabitants of the neighbourhood in which it is placed from any risk of infection, and at the same time, were the buildings sold, it would place the Children's Hospital in funds which they greatly need at the present time. Further steps should be taken to secure adequate convalescent accommodation for the patients treated at all the institutions, that at present provided being unsatisfactory and too limited in extent.

### Aberdeen's Opportunity.

Aberdeen's opportunity consists in a wise and prompt recognition of the advantages of the present position. With a reconstructed Infirmary, the erection of an adequate and extensive Nurses' Home, the placing of a staff of district nurses in the Dispensary buildings and the organisation of their work under the Dispensary medical officers, the throwing open of the Hospital for Incurables to the whole country, the restriction of out-patients to urgent cases sent in by the Dispensary medical officers with the addition of similar cases from the country districts, the reorganisation of the City Fever Hospital so as to provide accommodation for all cases of infectious diseases whether in children or adults, Aberdeen would indeed become an ideal city so far as its medical institutions are concerned. The overlapping of charity and the pauperisation of the population through the medical institutions would be prevented. The poor would have the enormous comfort of adequate hospital care in the severest cases, and of tender nursing and home comforts when overtaken by illness capable of being dealt with in their own homes. Infectious diseases might be reduced to a minimum, and every incurable case would receive such a full measure of attention that the suffering and care so often inseparable from the lot of this class of patients would be reduced to a minimum. In such circumstances the whole country might well be expected to come to Aberdeen in order to study its system of medical relief and its institutions, and if it should come to pass that the whole of the medical staff attached to the Infirmary and other medical institutions are to be paid for their services as well as those attached to the Dispensary, then Aberdeen would offer an example to gladden the heart and strengthen the hand of the wisest and most beneficent social reformers of our age and generation. We have had such cause to be proud of the intelligence and courage of Aberdonians in recent years that we make no doubt that within a reasonable time the ideal we have here ventured to sketch in outline may yet become a practical reality, pregnant for good to the whole population of the United Kingdom.

